# 

**Published:** 1851-02

**Authors:** 


### CONTENTS

### OF NO. II.

---

### ORIGINAL COMMUNICATIONS.

### ESSAYS AND CASES.

Art. I.—General Remarks on Alterative Medicines. By W. S. Chipley, M.D., of Lexington, Ky. - - - 93

Art. II.—Inverted Toe Nail: A new Remedy. By Benj. P. Drake, M.D., of Lexington, Ky. - - - - - 104

Art. III.—A Case of Tetanus. Reported by W. H. Cobb, M.D., of Louisville, Ky. - - - - 105

Art. IV.—A Case of Traumatic Tetanus, and recovery. By Theodore S. Bell, M.D., of Louisville, Ky. - 112

Art. V.—Convulsions; and the remarkable effects of Chloroform. By Theodore S. Bell, M.D. - - - 116

### SELECTIONS FROM FOREIGN AND HOME JOURNALS.

On the Present Condition and Treatment of Venereal Diseases in Paris. - - - - - - - 120

Epilepsy with Spectral Illusions. - - - - 129

A cheap and simple article of Nourishment for Infants. - 132

Treatment of Cardiac Dropsy. - - - - - 134

On the Diagnosis of Tape-Worm. - - - - - 141

Gonorrhea treated with the Acetate of Potash. - - 145

The Anthelmintic—Banksia Abyssinica or Kousso. - - 146

On the influence of Large Doses of Quinine in Neuralgia. - 148

On a Death from Heart Disease, occurring in a swimming bath. 150

Citrate of Caffeine in Hemicrania. - - - - - - 152

Success of the Kousso in the Expulsion of Tape-Worm. - 152
Anatomical Lucubrations at the Cape of Good Hope. - - 153
Practical illustrations of the Remedial Efficacy of a very low or Anæsthetic Temperature in Cancer. - - - 154

BIBLIOGRAPHICAL NOTICES.

Report of the Eighth General Meeting of the Sydenham Society, held at the Society's Rooms, on Wednesday, May 1st, 1850. Sir James Clark, Bart., M.D., F.R.S., President of the Society in the chair. - - - - - 161
Researches upon the Necropolis of New Orleans, with brief allusions to its Vital Arithmetic. By Bennet Dowler, M.D., of New Orleans. - - - - - - - 168

RETROSPECT OF AMERICAN MEDICAL INTELLIGENCE.

The Speculum Uteri. - - - - - - 163
Tetanus with recovery. - - - - - 175
Hydrocele. - - - - - - - 175
Ascites. - - - - - - - - 175
Dr. Davis's History of Medicine. - - - - 175
Chloroform an Antiseptic. - - - - - 176
On the Use of Therapeutic Agents in relieving Rigidity of the Os and Cervix Uteri. - - - - - - 177
Professor Paul F. Eve. - - - - - - 179
Professor Silliman. - - - - - - - 180
Auvert's Plates. - - - - - - - 180
Jarvis's Adjuster. - - - - - - - 182
American Medical Association. - - - - - 184

